# Supplementary figures and images for: Anonymous group structure algorithm based on community structure
Source: PeerJ Comput Sci. 2024 Sep 18;10:e2244. doi: 10.7717/peerj-cs.2244 (PMC11419626; doi:10.7717/peerj-cs.2244)

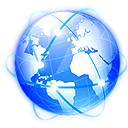

Supplement: Supplemental Information 1 [file peerj-cs-10-2244-s001.zip › Code/python/logo.jpg]

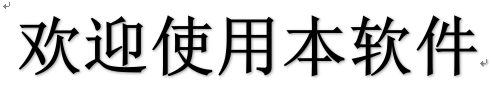

Supplement: Supplemental Information 1 [file peerj-cs-10-2244-s001.zip › Code/image/title.png]

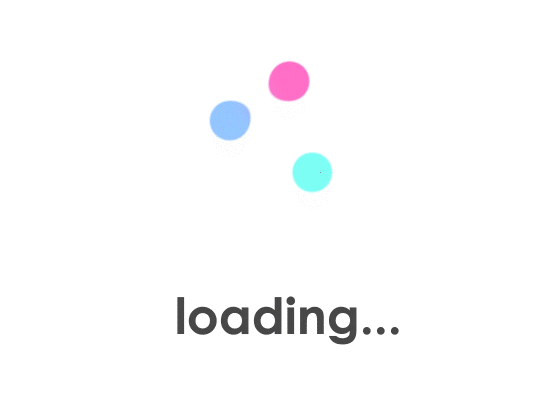

Supplement: Supplemental Information 1 [file peerj-cs-10-2244-s001.zip › Code/image/loading.gif]
